# Lack of advantages of slit mesh placement during laparoscopic transabdominal preperitoneal inguinal hernia repair (TAPP): a single centre, case matched study

**DOI:** 10.1186/s12893-018-0409-0

**Published:** 2018-09-20

**Authors:** Umberto Bracale, Jacopo Andreuccetti, Maurizio Sodo, Giovanni Merola, Giusto Pignata

**Affiliations:** 1 Department of General and Mini-invasive Surgery, San Camillo Hospital of Trento, Via Giovanelli 19, 38121 Trento, Italy; 2 Department of Surgical Specialities and nephrology, University Federico II Naples, Via Pansini 5, 80131 Naples, Italy

**Keywords:** Laparoscopy, No slit vs slit Mesh, Inguinal hernia repair, TAPP, Laparoscopic hernia repair

## Abstract

**Background:**

During laparoscopic trans-abdominal pre-peritoneal hernia repair (TAPP) the positioning of the mesh around the spermatic cord could provide an additional anchoring point and ensure better defect closure, thereby preventing mesh movement and recurrence. The primary aim of our retrospective study was to determine if, during a TAPP procedure, an advantageous difference for mesh placement exists between the slit and the non-slit techniques in terms of recurrence rate. Secondary aims were intra and post-operative complications and the time required to return to normal activity.

**Methods:**

From January 2010 to December 2015, data from patients who had undergone TAPPs at our Institution were prospectively collected. We performed a retrospective case control matched study of two homogenous (BMI, Age, type of hernia) groups of 100 patients who underwent respectively TAPP with no slit mesh placement (Group NS) and slit mesh placement (Group S). Statistical analysis was carried out using a SPSS 20. To compare continuous variables, an independent sample T-test was performed. A Chi-square test was employed for categorical data.

**Results:**

No differences were found between the slit and non-slit groups in terms of biometric features and intra and post-operative outcomes were found to be similar in both groups as well. In particular, at mean follow-up of 57.34 ± 10.56 months for Group NS and 55.66 ± 8.61 months for Group S months only one recurrence per group was found.

**Conclusion:**

Our study failed to prove a superiority of the slit mesh technique over the no-slit mesh technique during TAPP. However, in light of its not being a randomized study, a subsequent, well-designed RCT would be desirable in order to better determine if the Slit mesh technique could prove to be advantageous enough to justify its routine use during the TAPP procedure.

## Background

Inguinal hernia repair (IHR) is one of the most common procedures in general surgery worldwide and can be performed with both an open or laparoscopic approach (LA). Every year more than 800,000 IHRs are performed in the United States alone [1]. LA has the added advantage of combining mesh inguinal hernia repair with a minimally invasive technique thereby proving less painful and enabling a faster return to work and daily activities as well as having better aesthetic outcome. As far as we are aware, no differences between the Transabdominal preperitoneal hernia repair (TAPP) and the Totally extra-peritoneal hernia repair (TEP) approach has ever been reported in published literature [2].

The International Endohernia Society (IEHS) guidelines indicate to use a macroporose lightweight mesh sized up to 10x15cm, non-fixed or fixed with fibrin glue. Some controversy, due to the lack of strong evidences, arises around the slitting or non-slitting of the mesh in order to wrap it around the spermatic cord during laparoscopic inguinal hernia repair (LHR) [3, 4]. Some surgeons presume that a slit mesh wrapped around the cord, thereby fashioning a new internal ring, could prevent a hernia recurrence which, however, could cause circumferential scarring with subsequent postoperative pain. No evidence exists on spermatic cord injury nor a reduction in the recurrence rate due to the slit procedure has been published [5–11]. To date, only nine studies have compared the results of slit vs. no-slit mesh techniques, six during a TEP [5, 6, 12–15] and three others during a TAPP [10, 11, 16].

The primary aim of our case matched retrospective study was to determine if, during a TAPP procedure, an advantageous difference for mesh placement exists between the slit and the non-slit techniques in terms of recurrence rate, secondary aims were intra and post-operative complications and the time required to return to normal activity.

## Methods

This retrospective analysis of data was approved by the internal ethical commitee (Protocol n° 71/2018 of San Camillo Hospital of Trento ethical committee), we performed a retrospective analysis of data from patients who had undergone a TAPP in the Department of General and Mini-invasive surgery of San Camillo Hospital in Trento from January 2010 to December 2015. During this period a total of 516 patients underwent TAPP, of these 297 with no slit mesh placement and 219 with slit mesh placement. In order to avoid bias only male patients were included in the analysis. After female patients exclusion there were 267 patients with no slit mesh placement and 199 with slit mesh placement. All patient before surgery signed an informed consent for the surgical procedure and data collection.

We performed a retrospective case control matched study of two homogenous (BMI, Age, type of hernia) groups of 100 patients who underwent respectively TAPP with no slit mesh placement (Group NS) and slit mesh placement (Group S).

Indications for a LHR were bilateral inguinal hernia, recurrent inguinal hernia and monolateral inguinal hernia in young or sportsmen patients.

Contraindications included patient preference for open repair, ASA IV, and previous prostatectomy.

All procedures were performed by three different surgeons who had completed their learning curve and each of them had done at least 150 laparoscopic inguinal hernia repairs before.

Data collected included: gender, age, American Society of Anaesthesiologists risk class (ASA), Body Mass Index (BMI), operative time (OT), type of hernia according to IEHS classification [17], conversion rate, intra and post-operative complications such as bleeding, seroma, wound infection, numbness (evaluated at 1 week, at 1 month, 6 months and permanent), chronic pain, recurrence rate, hospital stay and time to return to normal activity. Chronic pain is defined as pain lasting 6 months or more [18–20]. Follow-up with clinical examination was conducted at 7 days, 1 month and every year from surgery.

### Statistical analysis

Statistical analysis was performed using IBM SPSS Statistics 23. The case matching was assessed only for biometrical features. Continuous data were expressed as mean ± Standard deviation (SD). To compare continuous variables, an independent sample T-test was performed. The Chi-square test was employed to analyse categorical data. All results are presented as 2-tailed values with statistical significance if *p* values < 0.05. Logistic regression was performed to assess if there were any correlation between complications and other factors.

### Perioperative management

Before surgery all patient had undergone a physical examination and a routine blood test only. In accordance with IEHS guidelines, antibiotics and thromboembolic prophylaxis were administered only in select cases [3]. An intraoperatively nasogastric tube and urinary catheter were placed and removed at the end of each procedure. Pain management was achieved by non-steroidal anti-inflammatory drugs (NSAIDs) which were administered when required. All patients resumed drinking in the evening following surgery. Most were discharged on the first postoperative day.

### Surgical technique

**Fig. 1:**
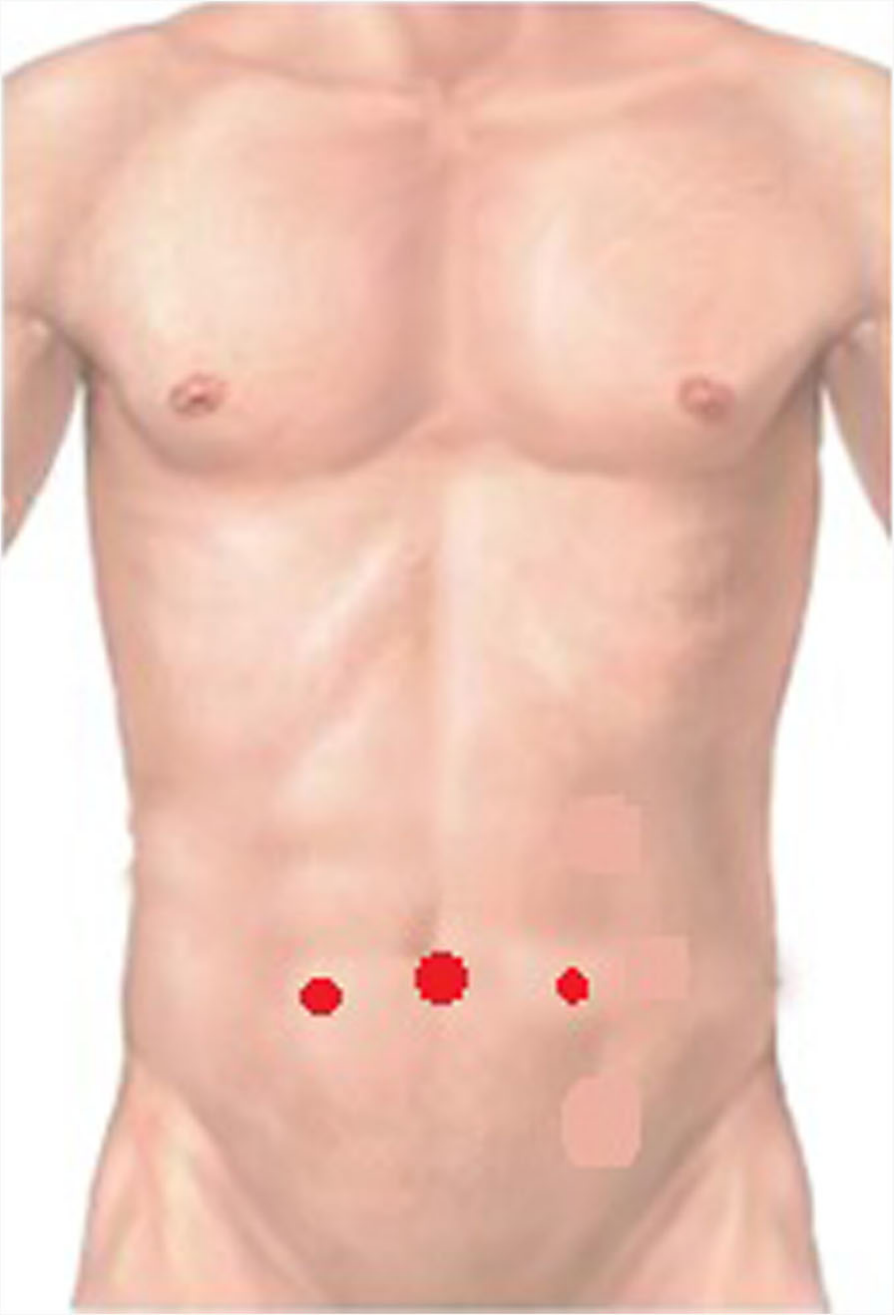
Trocars Position

The surgical technique for laparoscopic inguinal hernia repair is the same described in our previous paper [21]. Under general anaesthesia the patient was placed in a Trendelenburg position (30°) with arms and legs adduced. Pneumoperitoneum was established with the Veress technique [22]. The first 10 mm trocar was placed in the umbilicus, and two other 5 mm ports were placed bilaterally in the midclavicular line 1 cm below the umbilical line (Fig. 1). After identification of the anatomical landmarks (Epigastric Vessels, Spermatic vessels, vas deferens or round ligament, urachus, iliac vessels and bladder), the preperitoneal space was opened incising the peritoneum transversely from the region of the umbilical artery laterally to the hernia defect. The dissection was carried out into the Retzius and Bogros (retroinguinal) spaces. The anatomical landmarks (epigastric vessels, Cooper and Gimbernat ligaments, the corona mortis and external iliac vessels) were then identified and well exposed. The sac dissection was performed carefully safeguarding the spermatic fascia and protecting the fragile parietal structures.

**Fig. 2:**
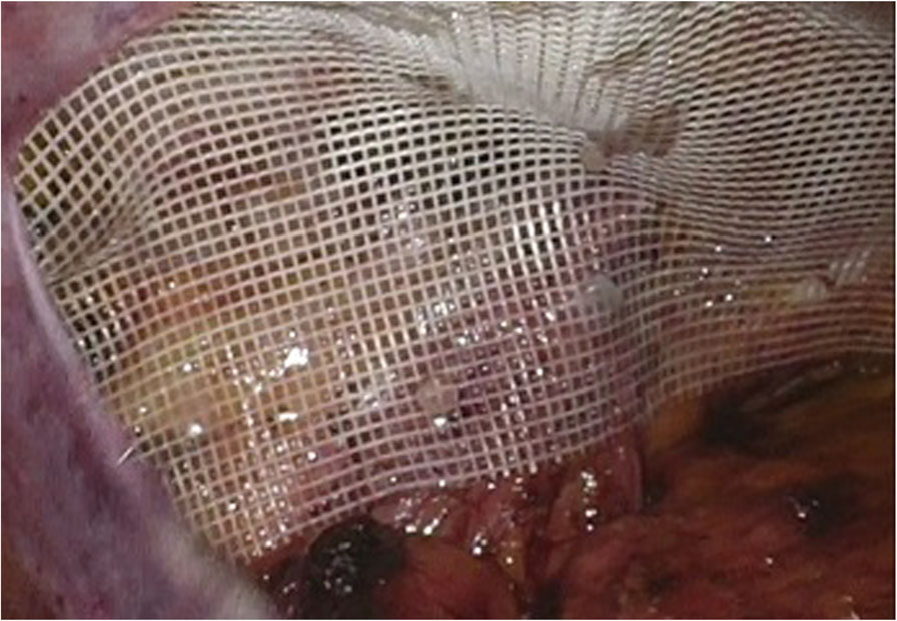
Non-Slit Mesh Placement

**Fig. 3:**
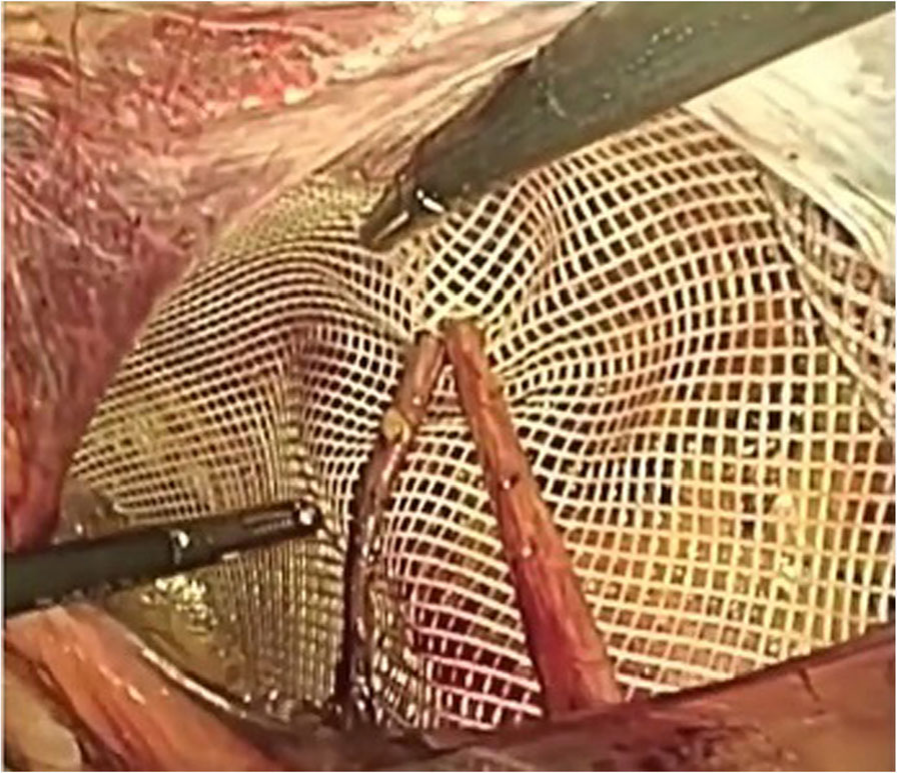
Slit Mesh Placement

A 10 × 15 cm polyester mesh was placed directly over the cord structures (Group NS) (Fig. 2), or “key-holed”, after engraving vertically the mesh in the upper part, to accommodate the cord (Group S) (Fig. 3). The mesh was securely fixed with 2 ml of fibrine glue (Tisseel/Tissucol, Baxter Healthcare, Deerfield, IL, USA) in order to avoid vessel injury or nerve entrapment, in accordance with IEHS guidelines [4]. The peritoneum was closed with a running suture in V-Loc 3/0, and trocars were removed under direct visualization. The fascial defect of 10 mm port was then closed under direct visualization.

## Results

No differences in biometric features existed between the two groups as reported in Table 1. As well, no variations in the distribution of unilateral and bilateral hernia between the two groups were noted such as that for the distribution of recurrent and primitive hernia. Intra and post-operative outcomes are reported in Table 2. Global operative time was comparable between the two groups. No statistical differences in OT for bilateral inguinal hernia was noted in either of the two groups (Group NS 85 ± 23.21 min; Group S 91.92 ± 22.75 min, *p* = 0.097) such as that noted for OT in unilateral inguinal hernia (Groups NS 77.2 ± 28.28 min; Group S 72.28 ± 17.59 min, *p* = 0.45). No conversions nor intraoperative complications had occurred in either group. Eleven and nine early, post-operative complications occurred in Group NS and Group S respectively. Seroma was the most frequent complication and only one bleeding and wound infection were found per group. The two bleeding reported (one per group) are of the navel as well as the two wound infections (one per group).

**Table 1:** Biometric Features

	Group NS 100pts	Group S 100pts	P
Age (Mean ± SD)	56.3 ± 12.01	58.17 ± 12.2	0.295
BMI (Mean ± SD)	25.53 ± 3.52	25.47 ± 3.77	0.912
Type of Hernia (N° Unilateral/Bilateral)	30/70	27/73	0.428
Type of Hernia (N° Primitive/Recurrent)	160/10	165/8	
Type of Hernia according to EHS classification			
*PL0M1F0*	9	7	
*PL0M2F0*	22	22	
*PL0M3F0*	12	10	
*PL1M2F0*	17	16	
*PL2M2F0*	9	11	
*PL3M2F0*	2	3	
*PL2M1F0*	2	1	
*PL1M3F0*	1	2	
*PL1M0F0*	29	31	
*PL2M0F0*	36	38	
*PL3M0F0*	8	10	
*PL0M0F1*	3	4	
*PL2M0F1*	4	5	
*PL2M0F2*	2	2	
*PL0M2F1*	4	3	
*RL2M0F0*	3	1	
*RL1M1F0*	1	1	
*RL0M1F0*	1	2	
*RL0M2F0*	2	1	
*RLOM3F0*	2	2	
*RL0M0F1*	1	1	
ASA Classification			
I	34	40	
II	48	45	
III	18	15

**Table 2:** Intra and Post-operative outcomes

	Group NS 100pts	Group S 100pts	P
Conversion Rate n°	0	0	1
Global Operative Time (Mean ± SD) in min	83.04 ± 24.99	87.01 ± 23.13	0.245
Operative Time in bilateral hernia (Mean ± SD) in min	85 ± 23.21	91.92 ± 22.75	0.097
Operative Time in unilateral hernia (Mean ± SD) in min.	77.2 ± 28.28	72.28 ± 17.59	0.45
Intraoperative complications n°	0	0	1
Post-Operative Complication			
Seroma (%)	9	7	0.602
Bleeding (%)	1	1	1
Wound Infection (%)	1	1	1
Hospital Stay (Mean ± SD) in days	1.04 ± 0.19	1.06 ± 0.23	0.519
Return to work in days (Mean ± SD)	15.88 ± 1.94	15.77 ± 0.23	0.718
Numbness			
1 week (%)	8	12	0.816
1 month (%)	2	4	0.407
6 months (%)	0	0	1
Permanent	0	0	1
Chronic Pain (%)	0	1	0.316
Recurrence (%)	1	1	1

No statistical difference was observed in length of hospital stay between the two groups and return to daily activity was comparable within both groups. No statistically significant difference occurred in recurrence, numbness and chronic pain between the groups. The median follow-up was 57.34 ± 10.56 months (Range 30–66 months) for Group NS and 55.66 ± 8.61 months (Range 24–64 months) for Group S (Table 2). We lost at follow-up 3 patients (3%) in group NS and 2 patients (2%) in group S.

## Discussion

Since 1990, when the first LHR repair was described, LA for treatment of groin hernia has been widely practiced [5]. TAPP and TEP techniques guarantee the same results as open inguinal hernia repair in terms of recurrence, which add to the advantages of LA over conventional surgery, such as less pain, faster return to daily activities and better aesthetic outcome. Some controversies arise around the slitting or not of the mesh employed in order to place it around the spermatic cord. Some surgeons presume that a slit mesh closed around the cord making a new internal ring, could prevent a recurrence albeit resulting in circumferential scarring with subsequent postoperative pain.

After a literature review, we found nine articles on the slit vs. non-slit topic [5, 6, 10–16] (Table 3). Leibl et Al. [10], in their three-arm randomized trial, reported no difference in the recurrence rate, OT, postoperative complications and pain killer assumption between their group A (employing a slit mesh) and the other two study groups (No slit mesh fixed with staples in group B or suture in group C). Their results found only one recurrence in Group C. Leibl et Al [23], also published a previous paper on the causes of inguinal hernia recurrence after TAPP, identifying incision of the mesh as a “second cause of recurrence”. However, in the above-mentioned trial, they concluded that mesh fashioning alternatives did not result in any differences in postoperative complaints or complications.

**Table 3:** Previous paspers about Slit VS non-Slit mesh placement technique

Author	Technique	Use of Slit	Number of patients in slit group	Recurrence (%)
*Velasco*	TAPP	Horizzontal slit	25	6
*Leibl 2002*	TAPP	Vertical slit	124	0
*Leibl 1998*	TAPP	Vertical slit	2700	1.03
*McKernan*	TEP	Vertical slit	34	?
*Phillips*	TEP	Oblique slit	172	0
*Chia*	TEP	Oblique slit	54	1.6
*Cristaudo*	TEP	NR	14	NR
*Celik*	TEP	Vertical slit	20	0
*Domniz*	TEP	Vertical slit	87	0.6

Trans abdominal preperitoneal hernia repair (TAPP); Totally extraperitoneal hernia repair (TEP), Not Reported (NR)

Leibl’s results are consistent with Celik et al.’s [5] previous study whereby they confirm that LHR has no negative effect on testicular blood flow and volume, nor on recurrence, in both Slit or No-Slit groups. Korman et al. [24], in their study on the fashioning of differently cut meshes, did not find any resulting differences in terms of recurrence and chronic pain.

Domniz et al. [6] evaluated the efficacy of slit mesh during TEP, reporting a statistically significant recurrence rate in favour of the slit group. They argued that their results, as a consequence of the wrapping of the mesh below the spermatic cord which provided an additional anchoring point and better defect closure, preventing mesh movement and recurrence. This hypothetical advantage could prove to be more effective in the presence of large indirect hernia. Domnniz’s group did find, however, that the slit mesh surgery took about 8 min longer than the non-slit procedure, implying that the Slit procedure could be a more challenging technique.

About the relatively high seroma rate found in both groups of our study, it could be explained for two reasons, in accordance with those reported in a recent study [25]. In the study period, we didn’t use any technical tricks to prevent the seroma development, as well as the ligation of Transversalis Fascia trough the Röder loop or the fixation of the Transversalis Fascia to the Cooper Ligament through a suture or through a not-absorbable tack. Secondarily, it is not negligible the rate of Large defects (L3 or M3) collected in our series. As reported by Köckerling et Al., a large hernia defect has a significantly higher risk of seroma formation. Finally, in all cases we had used the fibrin glue as fixation method, which presents a higher risk for seroma development in TAPP inguinal hernia repair [25].

Although the aim of our study did not commit the testis vascularization, no differences in inguinal or scrotal long-term numbness, nor testicular atrophy between the two groups were noted. Only a greater numbness in Group S (Group NS: 8; Group S: 17, p 0.054) during the first post-operative week was found. These results may be due to the greater surgical stress of the genital branch of the genito-femoral nerve during preparation of the spermatic cord structures yet the degree of numbness noted at 1 month was similar amongst the two groups. Similarly, no statistically significant differences in recurrence and chronic pain between the two groups was noted.

Although no consensus was found in the literature, IEHS recommends (Grade B) not cutting the mesh in order to allow for passage of the spermatic cord structures. This is because this technique does not necessarily reduce the recurrence rate and may cause injury to the funicle structures [4]. This recommendation is not supported by strong evidence concerning suspected risk of funicle structure injury nor postoperative testicular atrophy; however, it could be a consequence of a lack of advantages in terms of recurrence prevention.

Our results are consistent with IEHS’ recommendation even if we did not find the use of a slit mesh less safe, more risky or more time consuming then the use of a no slit mesh. Also, the recurrence rate in the subgroup of lateral defects in which the indication of a slit mesh would be more indicated, was not found to be different to that of the other subgroups (medial or femoral defects).

## Conclusion

In conclusion, our study, did not demonstrate the superiority of the slit mesh technique over the non-slit mesh technique in a TAPP. For this reason, a well-designed RCT is desirable in order to clarify if the Slit mesh technique has any advantages over the non-Slit technique to justify its routine employment in transabdominal inguinal hernia repair.

## Abbreviations

ASA: American Society of Anaesthesiologists risk class
BMI: Body Mass Index
IEHS: International endo hernia society
IHR: Inguinal hernia repair
LA: Laparoscopic approach
LHR: Laparoscopic hernia repair
NS: Non- slit
OT: Operative time
RCT: Randomized controlled trial
S: Slit
SD: Standard deviation
TAPP: Trans abdominal pre peritoneal hernia repair
TEP: Totally extra peritoneal hernia repair
